# Crystal structure and halogen–hydrogen bonding of a Delépine reaction inter­mediate

**DOI:** 10.1107/S2056989020015601

**Published:** 2021-01-01

**Authors:** David Z. T. Mulrooney, Helge Müller-Bunz, Tony D. Keene

**Affiliations:** a School of Chemistry, University College Dublin, Belfield, Dublin 4, Ireland

**Keywords:** crystal structure, Hirshfeld surface, Delépine reaction, urotropinium

## Abstract

The reaction of 1,5-di­bromo­pentane with urotropine results in crystals of the title mol­ecular salt, 5-bromo­urotropinium bromide [systematic name: 1-(5-bro­mo­pent­yl)-3,5,7-tri­aza-1-azoniatri­cyclo­[3.3.1.1^3,7^]decane bromide], C_11_H_22_BrN_4_^+^·Br^−^ (**1**), crystallizing in space group *P*2_1_/*n*. The packing in compound **1** is directed mainly by H⋯H van der Waals inter­actions and C—H⋯Br hydrogen bonds, as revealed by Hirshfeld surface analysis. Comparison with literature examples of alkyl­urotropinium halides shows that the inter­actions in **1** are consistent with those in other bromides and simple chloride and iodide species.

## Abstract 2

The reaction of 1,5-di­bromo­pentane with urotropine results in crystals of the title mol­ecular salt, 5-bromo­urotropinium bromide, crystallizing in space group *P*2_1_/*n*. The packing is directed mainly by H⋯H van der Waals inter­actions and C—H⋯Br hydrogen bonds, as revealed by Hirshfeld surface analysis.

## Chemical context   

Urotropine, C_6_H_12_N_4_ (also known as hexa­methyl­ene­tetra­mine, hmta) and its salts are widely used in chemical organic synthesis (Blažević *et al.*, 1979 ▸), as precursors for explosives (Fried *et al.*, 2001 ▸) and as pharmaceuticals (Lo *et al.*, 2014 ▸).

The Delépine reaction is a classic synthetic route to produce primary amines (Delépine, 1895 ▸, 1897 ▸). Alkyl or aryl halides are reacted with hmta to form a quaternary ammonium salt, followed by acid hydrolysis to give a primary amine. A major advantage of this reaction over other routes is that the formation of the quaternary urotropinium cation prevents further alkyl­ation and high yields are possible (Galat & Elion, 1939 ▸). We recently made an attempt to find a cost-effective route to synthesize 1,5-di­amino­pentane (cadaverine) from 1,5-di­bromo­pentane. On an industrial scale, this is produced by bacterial deca­rboxylation of lysine (Ma *et al.* 2017 ▸; Wang *et al.*, 2018 ▸). Attempts to react 1,5-di­bromo­pentane with hmta in the presence of NaI in ethanol (modified from Galat & Elion, 1939 ▸) led to the crystallization of a monosubstituted product, 5-bromo­urotropinium bromide, C_11_H_22_BrN_4_^+^·Br^−^ (**1**), the structure and supra­molecular features of which are presented here.
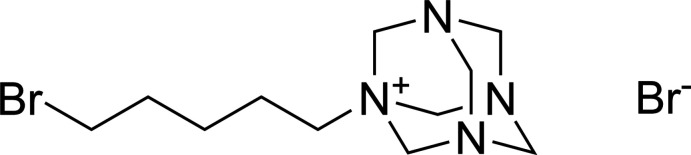


## Structural commentary   

**Figure 1:**
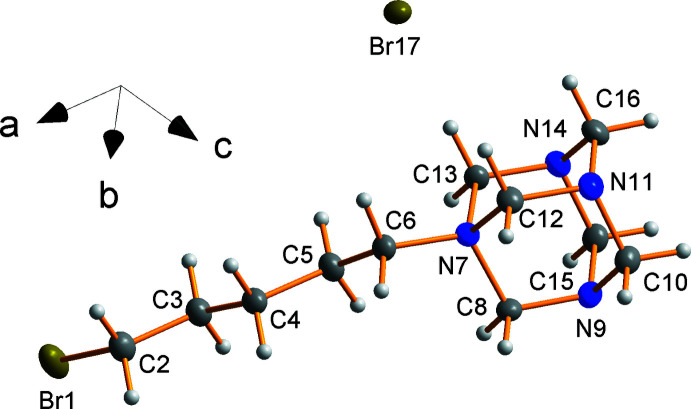
Asymmetric unit of compound **1**. Hydrogen atom labels are omitted for clarity. Displacement ellipsoids are at the 50% probability level.

Compound **1** crystallizes in the centrosymmetric monoclinic space group *P*2_1_/*n*. The asymmetric unit of **1** (Fig. 1 ▸) contains one C_11_H_22_BrN_4_^+^ *N*-(5-bromo­pent­yl)urotropinium cation and one bromide anion. The pentyl chain is in the all-*trans* configuration, unlike its hexyl relative (Reddy *et al.*, 1994 ▸), which displays an anti­clinal configuration between C4 and C6 of the hexyl chain (torsion angle = 133°).

## Supra­molecular features   

**Figure 2:**
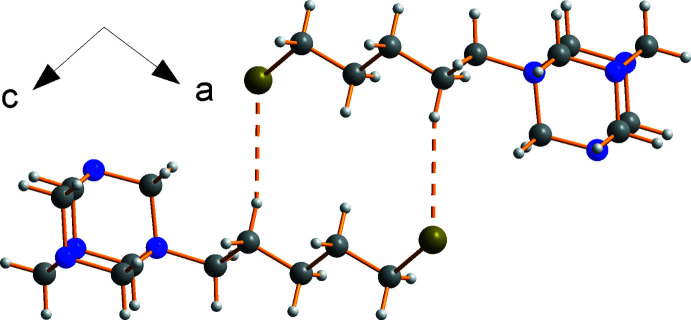
Dimerization of 5-bromo­pentyl­urotropinium cations through C—H⋯Br—C bonds.

The three-dimensional structure of **1** features C—H⋯Br^−^ and C—H⋯Br—C inter­actions (Table 1 ▸). Hirshfeld surface analysis of the urotropinium cation (see below for further details) reveals that the bromide anion, Br17, accepts C—H⋯Br hydrogen bonds from H12*A* and H13*B* [H⋯Br = 2.91 and 2.77 Å, respectively] within the asymmetric unit and forms bonds to H6*B*(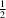 + *x*, 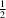 − *y*, −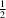 + *z*, 2.92 Å), H8*B* (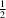 − *x*, −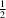 + *y*, 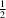 − *z*, 2.82 Å) and H15*A*(−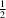 + *x*, 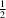 − *y*, −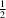 + *z*, 2.86 Å). C—H⋯Br—C bonds are also seen from H5*B* to Br1(1 − *x*, 1 − *y*, −*z*) at the end of the pentyl chain, linking neighbouring cations into an inversion dimer with an H⋯Br distance of 3.00 Å (Fig. 2 ▸).

**Figure 3:**
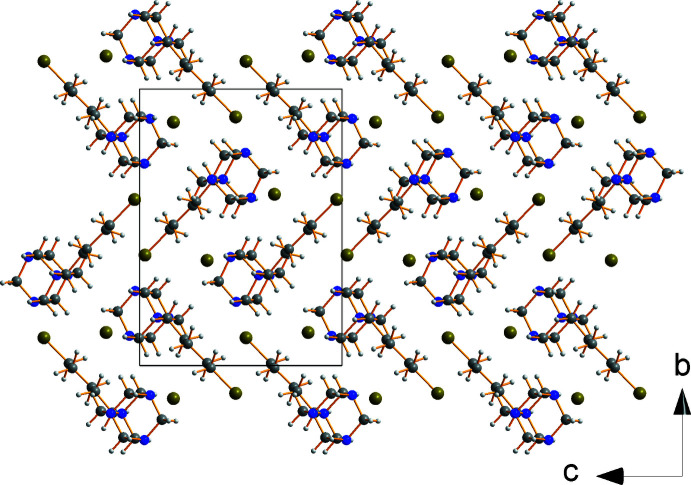
View of the crystal packing of compound **1** looking down the *a* axis.

The overall packing (Fig. 3 ▸) is similar to the hexyl compound.

## Hirshfeld surface analysis and two-dimensional fingerprint plots   

**Figure 4:**
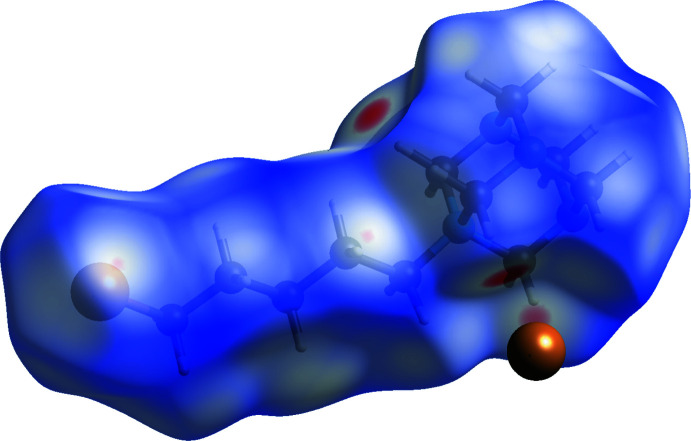
Hirshfeld surface of the 5-bromo­pentyl­urotropinium cation in compound **1** with the bromide anion shown. Surface plotted for *d*­_norm_ in the range −0.1755 to 1.3045 a.u.

**Figure 5:**
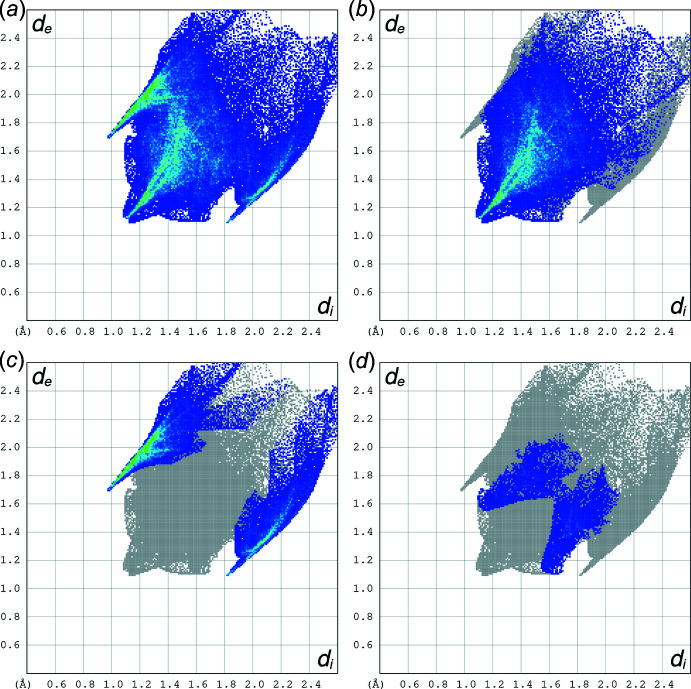
Two-dimensional fingerprint plots for compound **1**, showing (*a*) all inter­actions, (*b*) H⋯H, (*c*) H⋯Br and (*d*) H⋯N subsets.

The Hirshfeld surface analysis (Spackman & Jayatilaka, 2009 ▸) and two-dimensional fingerprint plots (McKinnon *et al.*, 2007 ▸) were calculated using *Crystal Explorer 17* (Turner *et al.*, 2017 ▸). The surface was calculated for the 5-bromo­pentyl­urotropinium cation (Fig. 4 ▸) in order to differentiate the contribution of the alkyl­bromine and bromide components to the overall bonding picture. In the *d*_norm_ plot (Fig. 5 ▸ *a*), white surface areas represent contacts at the sum of van der Waals radii, red is shorter (close contact) and blue is longer (long contact).

The Hirshfeld surface primarily consists of H⋯H van der Waals inter­actions (59.6%, Fig. 5 ▸ *b*) with the next major contributor being H⋯Br (31.0%, Fig. 5 ▸ *c*) with a small N⋯H component (9.4%, Fig. 5 ▸ *d*). The inter­action of H and the alkyl­bromine residue accounts for 12.6% of the surface (bottom right of Fig. 5 ▸ *c*), leaving the remaining 18.4% as H⋯Br^−^ hydrogen bonds to the bromide ions (top left of Fig. 5 ▸ *c*), as detailed above.

## Database survey   

Surprisingly few discrete alkyl­urotropinium salts have been submitted to the Cambridge Structural Database (version 5.41, May 2020 update 2, Groom *et al.*, 2016 ▸), given that the salts are reported to crystallize in most cases. There are 48 in total, 17 of which are halide or polyiodide salts. In the remainder, the alkyl­urotropinium exists as a counter-ion to complex anions or is a bridging species in a coordination polymer. Of the 17, only six are bromide salts (refcode BUXZEZ, Qingchuan *et al.* 1983 ▸; CAQVUO, Aniol *et al.*, 2017 ▸; CEXLOG, Mak, 1984 ▸; GINHAN, Betz & Klüfers, 2007 ▸; YOYWEO and YOYWIS, Reddy *et al.*, 1994 ▸).

A close relative to compound **1** is 6-bromo­hexyl­urotropinium bromide, C_12_H_24_BrN_4_^+^·Br^−^ (YOYWIS; Reddy *et al.*, 1994 ▸), which is isostructural, also crystallizing in space group *P*2_1_/*n*. As mentioned above, this displays an anti­clinal torsion angle in the alkyl chain, but presents very similar H⋯Br^−^ inter­actions and overall packing. For the purposes of comparison, the partial structure CIF in the CSD was completed in *OLEX2* to add in the hydrogen atoms present in the original publication, and Hirshfeld surface analysis also undertaken. A potential difficulty in this structure is the presence of a possible disorder in the hexyl chain (atom C10 has a markedly larger *U*_eq_ value than its neighbours, plus hydrogen atoms on C10 come into closer than van der Waals contact with hydrogen atoms on neighbouring C10 atoms in the crystal). However, the inter­actions between hydrogen and bromide account for a similar percentage of the overall Hirshfeld surface (16.6% in YOYWIS *versus* 18.4% in compound **1**).

Direct comparisons with BUXZEZ (Yang *et al.*, 1983 ▸) are difficult because of the disorder around the allyl group while the remaining compounds have other significant inter­molecular inter­actions such as hydrogen bonds formed to bromide by a water mol­ecule (CEXLOG, GINHAN) or from a carb­oxy­lic acid (YOYWEO). Similar inter­actions to compound **1** can be seen where chloride is the halide anion: BIDBIZ (Shao *et al.*, 1982 ▸) shows hydrogen bonds from the benzyl­urotropinium cation to the chloride anion, accounting for 12.2% of the inter­action surface. Polyiodide compounds appear not to show C—H⋯I inter­actions in the same manner as the above bromide and chloride compounds, but a methyl­urotropinium monoiodide compound (VOBCIY; Ribár *et al.*, 1991 ▸) displays similar inter­actions to **1** with H⋯I hydrogen bonds forming 15.5% of the Hirshfeld surface.

## Synthesis and crystallization   

Uroptropine (11.0 mmol, 1.542 g) and NaI (90.95 mmol, 1.648 g) were dissolved in ethanol and 1,5-di­bromo­pentane (5.00 mmol, 0.595 ml) was added. Clear block-like crystals appeared after 8 days, which were found to be a mixture of compound **1** and [Na(H_2_O)_4_(hmta)]_2_Br_2_·2H_2_O (Kruszynski *et al.*, 2012 ▸), which precluded further analysis, given the instability of hmta adducts to recrystallization.

## Refinement   

Crystal data, data collection and structure refinement details are summarized in Table 2 ▸. The H atoms were positioned geometrically (C—H = 0.99 Å) and refined as riding atoms with *U*_iso_(H) = 1.2*U*_eq_(C).

## Supplementary Material

Crystal structure: contains datablock(s) I. DOI: 10.1107/S2056989020015601/hb7951sup1.cif

Structure factors: contains datablock(s) I. DOI: 10.1107/S2056989020015601/hb7951Isup2.hkl

Supporting information file. DOI: 10.1107/S2056989020015601/hb7951Isup3.cml

CCDC reference: 2046576

Additional supporting information:  crystallographic information; 3D view; checkCIF report

## Figures and Tables

**Table 1:** Hydrogen-bond geometry (Å, °)

*D*—H⋯*A*	*D*—H	H⋯*A*	*D*⋯*A*	*D*—H⋯*A*
C12—H12*A*⋯Br17	0.99	2.91	3.800 (2)	150
C13—H13*B*⋯Br17	0.99	2.77	3.689 (2)	155
C15—H15*A*⋯Br17^i^	0.99	2.92	3.844 (3)	156
C8—H8*B*⋯Br17^ii^	0.99	2.82	3.777 (2)	162
C6—H6*B*⋯Br17^iii^	0.99	2.86	3.819 (2)	163
C5—H5*B*⋯Br1^iv^	0.99	3.00	3.960 (3)	163

Symmetry codes: (i) 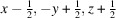; (ii) 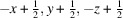; (iii) 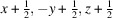; (iv) 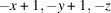.

**Table 2:** Experimental details

Crystal data	
Chemical formula	C_11_H_22_BrN_4_^+^·Br^−^
*M*_r_	370.14
Crystal system, space group	Monoclinic, *P*2_1_/*n*
Temperature (K)	100
*a*, *b*, *c* (Å)	8.7897 (2), 14.8937 (3), 11.1294 (2)
β (°)	101.742 (2)
*V* (Å^3^)	1426.47 (5)
*Z*	4
Radiation type	Cu *K*α
μ (mm^−1^)	7.09
Crystal size (mm)	0.08 × 0.06 × 0.05

Data collection	
Diffractometer	Rigaku Oxford Diffraction SuperNova, Dual, Cu at zero, Atlas
Absorption correction	Gaussian (*CrysAlis PRO*; Rigaku OD, 2015 ▸)
*T*_min_, *T*_max_	0.698, 0.788
No. of measured, independent and observed [*I* > 2σ(*I*)] reflections	22370, 2992, 2652
*R*_int_	0.040
(sin θ/λ)_max_ (Å^−1^)	0.632

Refinement	
*R*[*F* ^2^ > 2σ(*F* ^2^)], *wR*(*F* ^2^), *S*	0.025, 0.063, 1.04
No. of reflections	2992
No. of parameters	154
H-atom treatment	H-atom parameters constrained
Δρ_max_, Δρ_min_ (e Å^−3^)	0.53, −0.64

Computer programs: *CrysAlis PRO* (Rigaku OD, 2015 ▸), *SHELXT* (Sheldrick, 2015*a* ▸), *SHELXL2018/3* (Sheldrick, 2015*b* ▸), *DIAMOND* (Brandenburg & Berndt, 1999 ▸) and *OLEX2* (Dolomanov *et al.*, 2009 ▸).

## supplementary crystallographic information

### Crystal data

**Table:**

C_11_H_22_BrN_4_^+^·Br^−^	*F*(000) = 744
*M**_r_* = 370.14	*D*_x_ = 1.724 Mg m^−^^3^
Monoclinic, *P*2_1_/*n*	Cu *K*α radiation, λ = 1.54184 Å
*a* = 8.7897 (2) Å	Cell parameters from 10133 reflections
*b* = 14.8937 (3) Å	θ = 5.0–76.7°
*c* = 11.1294 (2) Å	µ = 7.09 mm^−^^1^
β = 101.742 (2)°	*T* = 100 K
*V* = 1426.47 (5) Å^3^	Block, colourless
*Z* = 4	0.08 × 0.06 × 0.05 mm

### Data collection

**Table:**

Rigaku Oxford Diffraction SuperNova, Dual, Cu at zero, Atlas diffractometer	2652 reflections with *I* > 2σ(*I*)
Detector resolution: 10.3196 pixels mm^-1^	*R*_int_ = 0.040
ω scans	θ_max_ = 76.9°, θ_min_ = 5.0°
Absorption correction: gaussian (CrysAlisPro; Rigaku OD, 2015)	*h* = −9→11
*T*_min_ = 0.698, *T*_max_ = 0.788	*k* = −18→17
22370 measured reflections	*l* = −13→14
2992 independent reflections	

### Refinement

**Table:**

Refinement on *F*^2^	Primary atom site location: dual
Least-squares matrix: full	Hydrogen site location: inferred from neighbouring sites
*R*[*F*^2^ > 2σ(*F*^2^)] = 0.025	H-atom parameters constrained
*wR*(*F*^2^) = 0.063	*w* = 1/[σ^2^(*F*_o_^2^) + (0.0253*P*)^2^ + 1.7861*P*] where *P* = (*F*_o_^2^ + 2*F*_c_^2^)/3
*S* = 1.04	(Δ/σ)_max_ = 0.001
2992 reflections	Δρ_max_ = 0.53 e Å^−^^3^
154 parameters	Δρ_min_ = −0.64 e Å^−^^3^
0 restraints	

### Special details

**Table:**

Geometry. All esds (except the esd in the dihedral angle between two l.s. planes) are estimated using the full covariance matrix. The cell esds are taken into account individually in the estimation of esds in distances, angles and torsion angles; correlations between esds in cell parameters are only used when they are defined by crystal symmetry. An approximate (isotropic) treatment of cell esds is used for estimating esds involving l.s. planes.

### Fractional atomic coordinates and isotropic or equivalent isotropic displacement parameters (Å^2^)

**Table:**

	*x*	*y*	*z*	*U*_iso_*/*U*_eq_	
Br17	0.26207 (3)	0.11960 (2)	0.16658 (2)	0.02528 (8)	
Br1	0.83401 (3)	0.60049 (2)	0.02566 (3)	0.03427 (9)	
N14	0.0484 (2)	0.32690 (14)	0.35699 (18)	0.0220 (4)	
N9	0.1817 (2)	0.39262 (14)	0.55095 (18)	0.0209 (4)	
N7	0.3320 (2)	0.32645 (13)	0.41148 (17)	0.0185 (4)	
N11	0.1807 (2)	0.23044 (14)	0.52273 (19)	0.0233 (4)	
C13	0.1875 (3)	0.33503 (16)	0.3076 (2)	0.0204 (4)	
H13A	0.188486	0.393964	0.266600	0.024*	
H13B	0.189079	0.287431	0.245757	0.024*	
C8	0.3233 (3)	0.40058 (15)	0.5049 (2)	0.0200 (4)	
H8A	0.413960	0.396114	0.573916	0.024*	
H8B	0.326628	0.459996	0.465655	0.024*	
C12	0.3222 (3)	0.23654 (16)	0.4761 (2)	0.0222 (5)	
H12A	0.325576	0.186875	0.417548	0.027*	
H12B	0.412836	0.230205	0.544750	0.027*	
C4	0.6585 (3)	0.42073 (17)	0.2631 (2)	0.0228 (5)	
H4A	0.723264	0.440004	0.342377	0.027*	
H4B	0.700757	0.363271	0.239322	0.027*	
C3	0.6661 (3)	0.49142 (17)	0.1656 (2)	0.0250 (5)	
H3A	0.601900	0.471674	0.086368	0.030*	
H3B	0.622209	0.548478	0.189060	0.030*	
C6	0.4807 (3)	0.32939 (16)	0.3650 (2)	0.0216 (5)	
H6A	0.491918	0.272273	0.322120	0.026*	
H6B	0.568745	0.333678	0.436101	0.026*	
C2	0.8312 (3)	0.50736 (18)	0.1499 (2)	0.0267 (5)	
H2A	0.874916	0.450893	0.124359	0.032*	
H2B	0.896396	0.526437	0.229127	0.032*	
C16	0.0478 (3)	0.23966 (17)	0.4192 (2)	0.0261 (5)	
H16A	−0.049980	0.233470	0.449665	0.031*	
H16B	0.051124	0.190783	0.359514	0.031*	
C5	0.4915 (3)	0.40688 (17)	0.2780 (2)	0.0241 (5)	
H5A	0.453165	0.462523	0.310466	0.029*	
H5B	0.424587	0.394188	0.196898	0.029*	
C15	0.0467 (3)	0.39888 (16)	0.4473 (2)	0.0228 (5)	
H15A	−0.050356	0.394968	0.479113	0.027*	
H15B	0.048158	0.457856	0.406527	0.027*	
C10	0.1763 (3)	0.30439 (16)	0.6095 (2)	0.0237 (5)	
H10A	0.265689	0.298837	0.679369	0.028*	
H10B	0.079868	0.299928	0.642209	0.028*	

### Atomic displacement parameters (Å^2^)

**Table:**

	*U*^11^	*U*^22^	*U*^33^	*U*^12^	*U*^13^	*U*^23^
Br17	0.02347 (13)	0.02523 (14)	0.02747 (13)	−0.00279 (9)	0.00593 (9)	−0.00790 (9)
Br1	0.02808 (14)	0.04267 (18)	0.03123 (15)	−0.00985 (11)	0.00409 (11)	0.00965 (11)
N14	0.0176 (9)	0.0237 (11)	0.0244 (10)	−0.0014 (7)	0.0038 (7)	0.0001 (8)
N9	0.0214 (9)	0.0215 (10)	0.0205 (9)	0.0016 (7)	0.0060 (7)	−0.0001 (7)
N7	0.0169 (9)	0.0173 (9)	0.0213 (9)	0.0007 (7)	0.0039 (7)	−0.0005 (7)
N11	0.0257 (10)	0.0193 (10)	0.0264 (10)	−0.0008 (8)	0.0092 (8)	0.0013 (8)
C13	0.0169 (10)	0.0237 (12)	0.0197 (10)	−0.0002 (8)	0.0018 (8)	−0.0002 (9)
C8	0.0212 (11)	0.0167 (11)	0.0220 (10)	−0.0005 (8)	0.0045 (8)	−0.0026 (8)
C12	0.0233 (11)	0.0167 (11)	0.0269 (12)	0.0019 (8)	0.0062 (9)	0.0013 (9)
C4	0.0191 (11)	0.0231 (12)	0.0268 (11)	0.0003 (9)	0.0063 (9)	−0.0024 (9)
C3	0.0218 (11)	0.0274 (13)	0.0264 (12)	−0.0019 (9)	0.0060 (9)	0.0011 (10)
C6	0.0178 (10)	0.0233 (12)	0.0248 (11)	0.0010 (8)	0.0069 (8)	−0.0023 (9)
C2	0.0248 (12)	0.0293 (13)	0.0264 (12)	−0.0019 (10)	0.0063 (9)	0.0037 (10)
C16	0.0233 (12)	0.0246 (13)	0.0308 (13)	−0.0067 (9)	0.0062 (10)	−0.0015 (10)
C5	0.0185 (11)	0.0273 (13)	0.0266 (12)	−0.0009 (9)	0.0050 (9)	0.0019 (9)
C15	0.0199 (11)	0.0239 (12)	0.0250 (11)	0.0049 (9)	0.0054 (9)	0.0012 (9)
C10	0.0269 (12)	0.0224 (12)	0.0230 (11)	0.0012 (9)	0.0077 (9)	0.0021 (9)

### Geometric parameters (Å, º)

**Table:**

Br1—C2	1.962 (3)	C4—H4A	0.9900
N14—C13	1.444 (3)	C4—H4B	0.9900
N14—C16	1.473 (3)	C4—C3	1.523 (3)
N14—C15	1.472 (3)	C4—C5	1.525 (3)
N9—C8	1.444 (3)	C3—H3A	0.9900
N9—C15	1.480 (3)	C3—H3B	0.9900
N9—C10	1.472 (3)	C3—C2	1.515 (3)
N7—C13	1.539 (3)	C6—H6A	0.9900
N7—C8	1.529 (3)	C6—H6B	0.9900
N7—C12	1.530 (3)	C6—C5	1.522 (3)
N7—C6	1.501 (3)	C2—H2A	0.9900
N11—C12	1.445 (3)	C2—H2B	0.9900
N11—C16	1.471 (3)	C16—H16A	0.9900
N11—C10	1.471 (3)	C16—H16B	0.9900
C13—H13A	0.9900	C5—H5A	0.9900
C13—H13B	0.9900	C5—H5B	0.9900
C8—H8A	0.9900	C15—H15A	0.9900
C8—H8B	0.9900	C15—H15B	0.9900
C12—H12A	0.9900	C10—H10A	0.9900
C12—H12B	0.9900	C10—H10B	0.9900
			
C13—N14—C16	109.72 (19)	H3A—C3—H3B	107.9
C13—N14—C15	109.01 (18)	C2—C3—C4	111.8 (2)
C15—N14—C16	108.66 (19)	C2—C3—H3A	109.2
C8—N9—C15	109.30 (18)	C2—C3—H3B	109.2
C8—N9—C10	109.79 (18)	N7—C6—H6A	108.7
C10—N9—C15	107.96 (19)	N7—C6—H6B	108.7
C8—N7—C13	107.64 (17)	N7—C6—C5	114.37 (19)
C8—N7—C12	107.27 (17)	H6A—C6—H6B	107.6
C12—N7—C13	107.99 (17)	C5—C6—H6A	108.7
C6—N7—C13	112.44 (17)	C5—C6—H6B	108.7
C6—N7—C8	112.39 (17)	Br1—C2—H2A	109.7
C6—N7—C12	108.89 (17)	Br1—C2—H2B	109.7
C12—N11—C16	108.50 (19)	C3—C2—Br1	109.96 (17)
C12—N11—C10	109.20 (19)	C3—C2—H2A	109.7
C10—N11—C16	108.76 (19)	C3—C2—H2B	109.7
N14—C13—N7	109.89 (18)	H2A—C2—H2B	108.2
N14—C13—H13A	109.7	N14—C16—H16A	109.2
N14—C13—H13B	109.7	N14—C16—H16B	109.2
N7—C13—H13A	109.7	N11—C16—N14	111.85 (19)
N7—C13—H13B	109.7	N11—C16—H16A	109.2
H13A—C13—H13B	108.2	N11—C16—H16B	109.2
N9—C8—N7	110.37 (18)	H16A—C16—H16B	107.9
N9—C8—H8A	109.6	C4—C5—H5A	109.4
N9—C8—H8B	109.6	C4—C5—H5B	109.4
N7—C8—H8A	109.6	C6—C5—C4	111.0 (2)
N7—C8—H8B	109.6	C6—C5—H5A	109.4
H8A—C8—H8B	108.1	C6—C5—H5B	109.4
N7—C12—H12A	109.4	H5A—C5—H5B	108.0
N7—C12—H12B	109.4	N14—C15—N9	111.64 (19)
N11—C12—N7	111.02 (18)	N14—C15—H15A	109.3
N11—C12—H12A	109.4	N14—C15—H15B	109.3
N11—C12—H12B	109.4	N9—C15—H15A	109.3
H12A—C12—H12B	108.0	N9—C15—H15B	109.3
H4A—C4—H4B	108.1	H15A—C15—H15B	108.0
C3—C4—H4A	109.5	N9—C10—H10A	109.3
C3—C4—H4B	109.5	N9—C10—H10B	109.3
C3—C4—C5	110.7 (2)	N11—C10—N9	111.71 (19)
C5—C4—H4A	109.5	N11—C10—H10A	109.3
C5—C4—H4B	109.5	N11—C10—H10B	109.3
C4—C3—H3A	109.2	H10A—C10—H10B	107.9
C4—C3—H3B	109.2		
			
N7—C6—C5—C4	164.35 (19)	C3—C4—C5—C6	173.8 (2)
C13—N14—C16—N11	61.4 (3)	C6—N7—C13—N14	176.92 (19)
C13—N14—C15—N9	−61.1 (2)	C6—N7—C8—N9	−177.46 (18)
C13—N7—C8—N9	58.2 (2)	C6—N7—C12—N11	179.94 (19)
C13—N7—C12—N11	−57.7 (2)	C16—N14—C13—N7	−58.7 (2)
C13—N7—C6—C5	49.2 (3)	C16—N14—C15—N9	58.4 (2)
C8—N9—C15—N14	60.4 (2)	C16—N11—C12—N7	59.3 (2)
C8—N9—C10—N11	−60.0 (2)	C16—N11—C10—N9	−58.7 (2)
C8—N7—C13—N14	−58.8 (2)	C5—C4—C3—C2	179.3 (2)
C8—N7—C12—N11	58.1 (2)	C15—N14—C13—N7	60.2 (2)
C8—N7—C6—C5	−72.4 (2)	C15—N14—C16—N11	−57.7 (2)
C12—N7—C13—N14	56.8 (2)	C15—N9—C8—N7	−58.9 (2)
C12—N7—C8—N9	−57.8 (2)	C15—N9—C10—N11	59.0 (2)
C12—N7—C6—C5	168.85 (19)	C10—N9—C8—N7	59.3 (2)
C12—N11—C16—N14	−60.9 (3)	C10—N9—C15—N14	−59.0 (2)
C12—N11—C10—N9	59.5 (2)	C10—N11—C12—N7	−59.1 (2)
C4—C3—C2—Br1	−178.93 (17)	C10—N11—C16—N14	57.8 (2)

### Hydrogen-bond geometry (Å, º)

**Table:**

*D*—H···*A*	*D*—H	H···*A*	*D*···*A*	*D*—H···*A*
C12—H12*A*···Br17	0.99	2.91	3.800 (2)	150
C13—H13*B*···Br17	0.99	2.77	3.689 (2)	155
C15—H15*A*···Br17^i^	0.99	2.92	3.844 (3)	156
C8—H8*B*···Br17^ii^	0.99	2.82	3.777 (2)	162
C6—H6*B*···Br17^iii^	0.99	2.86	3.819 (2)	163
C5—H5*B*···Br1^iv^	0.99	3.00	3.960 (3)	163

Symmetry codes: (i) *x*−1/2, −*y*+1/2, *z*+1/2; (ii) −*x*+1/2, *y*+1/2, −*z*+1/2; (iii) *x*+1/2, −*y*+1/2, *z*+1/2; (iv) −*x*+1, −*y*+1, −*z*.
